# 938 Impact of Preexisting Substance Use Disorders on Prolonged Opioid Use and Postoperative Complications After Burns

**DOI:** 10.1093/jbcr/iraf019.469

**Published:** 2025-04-01

**Authors:** Philong Nguyen, Joshua Lewis, Isabelle Carroll, Mbinui Ghogomu, Blancheneige Beohon, Matthew Dao, Amina El Ayadi, Steven Wolf, Juquan Song

**Affiliations:** University of Texas Medical Branch; University of Texas Medical Branch; University of Texas Southwestern Medical Center; University of Texas Medical Branch; University of Texas Medical Branch; University of Texas Medical Branch; University of Texas Medical Branch; University of Texas Medical Branch; University of Texas Medical Branch

## Abstract

**Introduction:**

Previous literature has highlighted the association between preexisting substance use disorders (SUDs) and adverse postoperative outcomes such as wound infections, postprocedural pain, and wound healing disruptions in burn patients. The impact of preexisting SUDs on prolonged opioid use and postoperative complications remains unclear. We hypothesize that preexisting substance use disorders (SUDs) increase the risk of opioid dependence and contribute to adverse postoperative complications in burn patients. This retrospective cohort study evaluates the effect of alcohol, cannabis, and tobacco SUDs on long-term opioid use and postoperative complications in adult burn patients.

**Methods:**

The TriNetX database was queried to identify adult burn patients (aged 18+) with preexisting diagnoses of alcohol, cannabis, or tobacco SUDs who sustained burn injuries. Patients were matched to a non-SUD cohort based on demographics, mental health conditions, pain syndromes, and burn severity using propensity score matching. Outcomes measured included prolonged opioid use and postoperative complications, such as wound infections, postprocedural pain, and wound healing disruptions. Risk ratios (RR) were calculated at 90 days and 1-year post-injury. Statistical significance was set at p < 0.05.

**Results:**

Before matching, 24,940 alcohol SUD, 20,274 cannabis SUD, and 77,543 tobacco SUD patients were identified with burn injuries. After matching, SUD patients had significantly higher risk ratios for prolonged opioid use at 90 days (Alcohol: RR 1.899, Cannabis: RR 1.832, Tobacco: RR 1.568) and 365 days (Alcohol: RR 1.798, Cannabis: RR 1.650, Tobacco: RR 1.581) compared to non-SUD patients. Similarly, the risk of wound infections was elevated at 90 days (Alcohol: RR 3.702, Cannabis: RR 2.864, Tobacco: RR 2.157) and 365 days (Alcohol: RR 2.452, Cannabis: RR 2.144, Tobacco: RR 1.898). SUD patients also exhibited increased risk of postprocedural pain at 90 days (Alcohol: RR 2.122, Cannabis: RR 2.307, Tobacco: RR 1.636) and 365 days (Alcohol: RR 1.761, Cannabis: RR 1.802, Tobacco: RR 1.578), as well as higher risks for wound healing disruptions at 90 days (Alcohol: RR 2.472, Cannabis: RR 2.906, Tobacco: RR 1.752) and 365 days (Alcohol: RR 2.001, Cannabis: RR 1.897, Tobacco: RR 1.555). All findings were statistically significant (p < 0.005).

**Conclusions:**

Burn patients with preexisting SUDs face a significantly higher risk of prolonged opioid use and postoperative complications. Burn patients with preexisting alcohol-related disorders showed the most significant increase in opioid use. Cannabis use was associated with the highest rate of postoperative complications at 3 and 12 months after burn.

**Applicability of Research to Practice:**

Personalized pain management and targeted SUD treatment protocols for burn patients are essential for mitigating opioid dependence, reducing the likelihood of complications, and enhancing long-term recovery outcomes.

**Funding for the Study:**

Supported by UTMB Institute for Translational Sciences (UL1 TR001439), funded by the National Center for Advancing Translational Sciences at the NIH.

